# Study on spin and optical polarization in a coupled InGaN/GaN quantum well and quantum dots structure

**DOI:** 10.1038/srep35597

**Published:** 2016-10-19

**Authors:** Jiadong Yu, Lai Wang, Di Yang, Jiyuan Zheng, Yuchen Xing, Zhibiao Hao, Yi Luo, Changzheng Sun, Yanjun Han, Bing Xiong, Jian Wang, Hongtao Li

**Affiliations:** 1 Tsinghua National Laboratory for Information Science and Technology, Department of Electronic Engineering, Tsinghua University, Beijing 100084, China

## Abstract

The spin and optical polarization based on a coupled InGaN/GaN quantum well (QW) and quantum dots (QDs) structure is investigated. In this structure, spin-electrons can be temporarily stored in QW, and spin injection from the QW into QDs via spin-conserved tunneling is enabled. Spin relaxation can be suppressed owing to the small energy difference between the initial state in the QW and the final states in the QDs. Photoluminescence (PL) and time-resolved photoluminescence (TRPL) measurements are carried out on optical spin-injection and -detection. Owing to the coupled structure, spin-conserved tunneling mechanism plays a significant role in preventing spin relaxation process. As a result, a higher circular polarization degree (CPD) (~49.1%) is achieved compared with conventional single layer of QDs structure. Moreover, spin relaxation time is also extended to about 2.43 ns due to the weaker state-filling effect. This coupled structure is believed an appropriate candidate for realization of spin-polarized light source.

The possibility to control electron spins in semiconductors has attracted a great attention for realization of spin-polarized light source, such as spin-polarized light-emitting diodes (spin-LEDs) and spin-polarized laser diodes (spin-LDs)^1^,^2^,^3^,^4^. There is a strong interest in using wide band-gap III-nitrides in spintronic applications, since the magnetic coupling strength is inversely dependent on lattice constant and the III-nitrides have been predicted to exhibit Curie temperatures above 300 K^5^,^6^. Furthermore, in order to obtain a long spin coherence time in semiconductors, the material must have weak spin-orbit interactions. Since GaN has a wider energy gap and also a much weaker spin-orbit interaction than GaAs, it would appear to be a natural candidate for spintronic devices^6^,^7^,^8^,^9^,^10^. Besides, the spin lifetime of carriers or excitons in InGaN quantum well (QW) and quantum dot (QD) is longer than that in bulk material due to the lifted degeneration of hole bands resulted from the spin-orbit splitting, crystal field splitting as well as the strong quantum confinement^11^,^12^. So the initial spin polarization of carriers or excitons can be more than 50% in the InGaN QW and QDs according to the “selection rules”^1^,^7^,^13^,^14^. Previous studies have been carried out on InGaN QW and InGaN QDs^2^,^6^,^15^,^16^,^17^. But there are still some challenges for single layer of InGaN QW or InGaN QDs structure to achieve a higher circular polarization degree (CPD, defined as (I_σ+_−I_σ−_)/(I_σ+_+I_σ−_)). Because of the influence of the spin relaxation effect^9^,^18^,^19^, spin polarization degree of electrons on conduction band will decrease. Thus, the CPD of PL will also decrease as follows^11^,^20^:


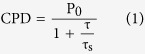


where P_0_ is the CPD without considering spin relaxation, τ is the carrier recombination lifetime, and τ_s_ is the spin relaxation time. For InGaN QW, quantum-confined Stark effect (QCSE) will lead to a relatively long radiative recombination lifetime and spin-polarized carriers will undergo more relaxation before recombination^21^,^22^, thus the CPD of the emission light will not be high. For InGaN QDs, although the QCSE can be weakened due to the smaller strain, polarized carriers generated in QDs will occupy the higher energy levels on account of the stronger quantum size effect^23^. The carriers on higher energy level (which have a long radiative recombination lifetime) will bear more serious spin relaxation before recombination, resulting in a lower initial spin polarization degree of carriers and hence a reduced CPD. In this paper, we report the spin and optical polarization based on a coupled InGaN/GaN QW and QDs structure^24^. In this structure, spin-electrons can be temporarily stored in QW, and injected from the QW into QDs via spin-conserved tunneling. Spin relaxation can be suppressed in this spin injection owing to the small energy difference between the initial state in QW and the final states in QDs^24^,^25^. So the radiative recombination mainly occurs in QDs to avoid the influence of strong QCSE in QW, and meanwhile, QW plays a role as a reservoir to avoid the carriers’ distribution in QDs too saturated. Besides, the strain within the QDs layer is weakened when it grown on InGaN QW^26^, hence the radiative recombination lifetime can be further reduced compared with the single layer of InGaN QDs structure. Therefore, this coupled structure, combining the advantages of both QW and QD, enables to achieve a higher CPD and a longer τ_s_ than single layer of QDs structure.

The schematic of spin injection in the coupled QW/QDs structure is shown in Fig. 1a. The spin-polarized electrons are generated in InGaN QW by σ^+^ polarized excitation light and then are injected into InGaN QDs through the phonon assisted tunneling^24^,^25^. It can be deduced by simulation that the energy difference between the ground state energy level (*E*_*W0*_) in QW and the first excited state energy level (*E*_*D1*_) in QDs is about 80–100 meV^18^, which nearly equals to a longitudinal optical (LO) phonon’s energy. So the electron can spatially transfer from *E*_*W0*_ into *E*_*D1*_ via phonon assisted tunneling and subsequent energy relaxation, rapidly emitting a longitudinal optical phonon and coupling with a few of acoustic phonons^25^. Since the radiative recombination lifetime of carriers in QD is short, a light with a certain CPD can be obtained before the electrons of majority spin state totally relax. Selection rules (see details in **Methods**) allowed radiative interband transitions in InGaN QW and InGaN QDs are shown in Fig. 1b,c, respectively, wherein epitaxial strain and quantum confinement have lifted the heavy- and light-hole band degeneracy. From the pictures, we can know that the transition probability in InGaN QW from heavy hole valence band (HH) to conduction band (CB) is over three times than that from light hole valence band (LH) to CB in the case of stimulated absorption. And the transition probability in InGaN QDs from CB to HH is also over three times than that of from CB to LH. Due to these factors along with the spin tunneling, PL with a high CPD can be obtained. Moreover, when the photon energy of excitation source is between HH and LH of QW, the CPD can be further improved.

The coupled QW-QDs sample, labeled as A, was grown by metal organic chemical vapor deposition (MOCVD). A 30-nm-thick low-temperature GaN buffer layer was grown on c-plane patterned sapphire substrates, followed with a 4-μm-thick undoped GaN layer, a 4.5-nm In_0.12_Ga_0.88_N QW, a 4.5-nm GaN barrier, a layer of In_0.3_Ga_0.7_N QDs, and a 9-nm GaN capping layer. The growth details can be found in **Methods**. For comparison, a sample with only single layer of QD as active region, labeled as B, was also grown, keeping all the other growth parameters the same as sample A. The characterization on structure was reported in our previous publication^23^. We performed optical spin-injection and -detection with photoluminescence (PL) and time-resolved photoluminescence (TRPL). The PL test system is schematically shown in Fig. 2 27. It is worth mentioning that the monochromator is sensitive to the polarization direction of linearly polarized lights. So a 532-nm quarter-wave plate (QWP) was used in front of the monochromator to convert linearly polarized light into a circularly polarized light.

## Results and Discussion

**Figure 3:**
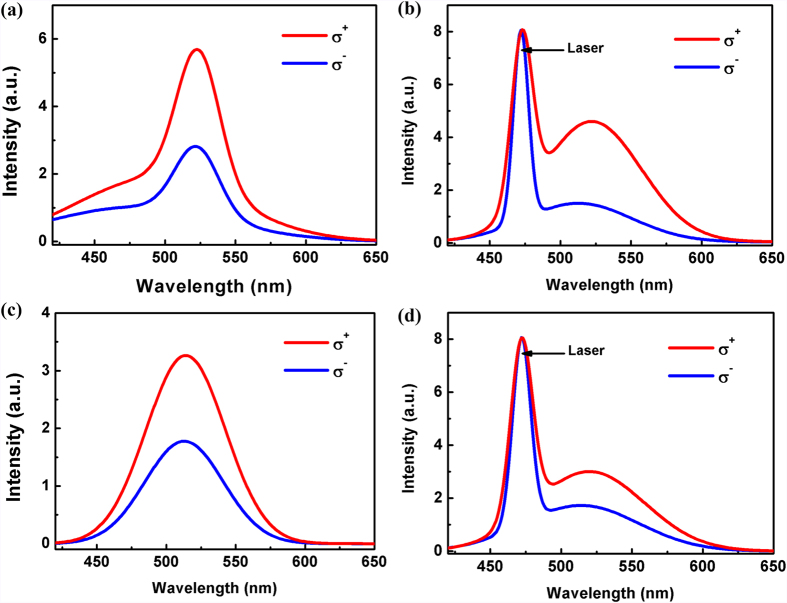
Circularly polarized PL spectra of (**a**) sample A under 405 nm excitation, (**b**) sample A under 473 nm excitation, (**c**) sample B under 405 nm excitation, and (**d**) sample B under 473 nm excitation. Both of the excitation power are 40 mW.

Figure 3a,d show circularly polarized PL spectra of samples A and B under 405 and 473 nm excitation, respectively. The excitation powers are both 40 mW. The QDs’ integral CPD of samples A and B are 33.7% and 23.7% under 405 nm excitation while 47.6% and 24.1% under 473 nm excitation, respectively. It can be seen that due to the spin-conserved tunneling, the CPD of QDs is increased in sample A. What’s more, the CPD of sample B almost keeps invariant while that of sample A increases significantly when the excitation wavelength changes from 405 nm to 473 nm. This phenomenon can be explained as follows. According to our previous result^24^, the excitation photon energy of 473 nm is corresponding to the energy value between HH to CB and LH to CB. So more electrons on HH are likely to be excited into the CB in this case. Because the transition probability from CB to HH is over three times than that from CB to LH, the CPD is further increased according to the “selection rules”. However, sample B doesn’t have such QW and its band edge of QDs is far away from the peak wavelength of these two excitation sources, hence the CPD still remains the similar value.

**Figure 4:**
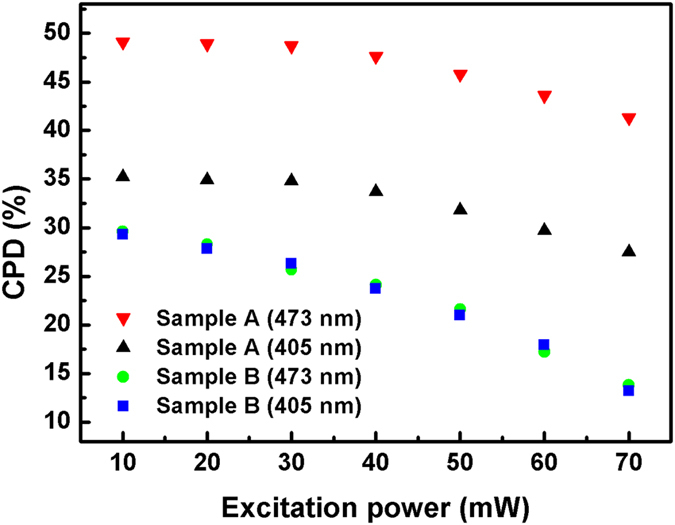
CPD obtained from PL test with variable excitation power of samples A and B. The excitation power increases from 10 mW to 70 mW with a step of 10 mW for both 405 and 473 nm sources, respectively.

In order to observe the influence of coupled structure on suppressing the state-filling effect more clearly, PL tests with variable excitation power are carried out on samples A and B as shown in Fig. 4. The excitation power increases from 10 mW to 70 mW with a step of 10 mW for both 405 and 473 nm sources, respectively. Figure 4 shows that the CPD of sample A hardly changes when the excitation power is lower than 30 mW (the highest CPD of 49.1% is achieved under 10 mW excitation), and decreases a little when the excitation power increases to 70 mW. This indicates that the state-filling effect in sample A is not serious. On the contrary, the CPD of sample B keeps decreasing as excitation increasing, implying its state-filling effect should be more serious. This phenomenon well reflects the advantage of the coupled structure that relieves the state-filling effect to some extent.

**Figure 5:**
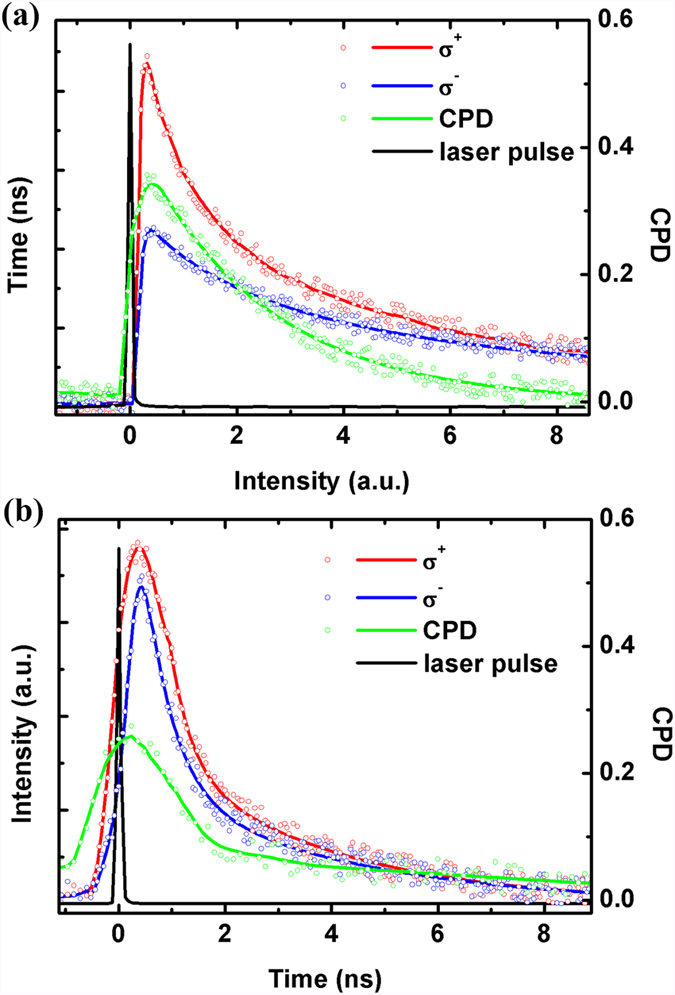
σ^+^- (red lines) and σ^−^- (blue lines) polarized TRPL spectra (**a**,**b**) and the corresponding CPD (green lines) for samples A and B, respectively.

The TRPL spectra of QDs experiment results are shown in Fig. 5a,b for samples A and B, respectively. For simplicity, the spin relaxation times of QDs are determined when CPD reduces to 1/*e* of its maximum value^24^,^28^, which are estimated to be 2.43 and 1.51 ns for samples A and B, respectively. Similarly, the carrier lifetime of samples A and B are estimated to be 1.44 and 1.28 ns, respectively. The longer carrier lifetime of sample A mainly results from the electrons tunneling from QW to QDs as well as the relief of carrier leakage in QDs. And the longer spin relaxation times of sample A is because of the weaker state-filling effect. Besides, both the PL intensity and CPD show a single-exponential decay in sample A while they show a double-exponential decay in sample B. Generally, there are two explanations on the double-exponential decay process. One is that there exist radiative and non-radiative recombination at the same time^29^,^30^,^31^. Due to the InGaN QW as a carrier reservoir, the carrier leakage and non-radiative recombination can be relieved in InGaN QDs. The other one is that the polarization field can lead to slower recombination velocity of a portion of the carriers^32^,^33^,^34^,^35^. The polarization field in the InGaN QDs layer of sample A is relatively weaker because of the smaller strain inside since they are grown on InGaN QW, which can be demonstrated through PL test with a variety of excitation power under 405 nm excitation (details can be found in Supplementary Information). No matter which one is the exact reason, therefore, the single-exponential decay in sample A is reasonable.

## Conclusion

In summary, a coupled InGaN/GaN QW-QDs structure is proposed to enhance the spin transfer and conservation. It demonstrates a higher circular polarization degree (CPD) (~49.1%), weaker state-filling effect and a longer spin relaxation time (~2.43 ns) compared with the single layer of QDs structure. The result indicates that the spin-conserved tunneling mechanism can play a significant role in preventing spin relaxation process and is promising to be applied in highly efficient spintronic devices.

## Methods

### Selection rules

Left- and right-circularly polarized photons have a projection ***m***_***j***_ of their angular momentum ***J*** on the direction of the wave vector equal to +*ℏ* (|↑>) or −*ℏ* (|↓>), respectively. The transition rate ***W***_***ij***_ can be calculated from the initial and final wave functions of the state involved in the transition using Fermi’s golden rule:


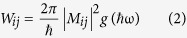


where 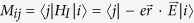 is the matrix element for the transition, and 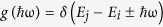 is the density of states. The matrix element describes the strength of the coupling between the initial and final states while the density of states represents the number of ways in which the transition can occur. For electronic states close to the Γ-point, the electron wave functions in the conduction and valence bands are well described by Bloch wave functions which have nearly the same orbital character as atomic states. The Bloch states may be denoted according to the total angular momentum, ***J***, and the projection of the total angular momentum onto the +z-axis, ***m***_***j***_, by |***J***, ***m***_***j***_ >. In this notation, the wave functions describing conduction and valence band states near the Γ-point may be expressed in terms of wave functions with X, Y, Z and S orbital character as shown in Table 1.

According to the selection rules, non-zero matrix elements (***M***_***ij***_ ≠ 0) are those for which ***Δm***_***j***_ = ±1. Along +z direction, a transition for which***Δm***_***j***_ = +1 leads to the emission of σ^+^-polarized light, while ***Δm***_***j***_ = −1 leads to the emission of σ^−^-polarized light. In the case of interband transitions between conduction band and valence band, the relative transition probabilities are determined by the square of the matrix element and are summarized in Table 2, given the spin part of the wave functions should be treated as <↑|↑> = <↓|↓> = 1 and <↑|↓> = <↓|↑> = 0. This selection rules only strictly valid at the Γ-point. But for spin and optical polarization character of spin-LEDs, it should be a very good approximation^36^.

According to Table 1 and Table 2 it can be seen that transitions including heavy hole are three times more probable than those including light holes. Considering the heavy hole and light hole valence bands are degenerate at the Γ-point in bulk GaN and InGaN, the relationship between spin polarization degree (***P***_***spin***_) and optical polarization degree (***P***_***opt***_) is given in the following equation:


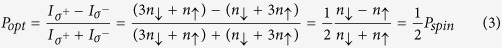


where I_σ+_ and I_σ−_ are the intensities of σ^+^ and σ^−^-polarized light, respectively. And n↑ and n↓ are the densities of spin-up and spin-down electrons. From Eq. 3 it can be seen that the ***P***_***opt***_ cannot exceed 50% in bulk materials (0 ≤ ***P***_***spin***_ ≤ 1).

However, in InGaN QW and QD, due to the spin-orbit splitting, crystal field splitting as well as the strong quantum confinement, the heavy and light hole valence bands are lifted near the Γ-point. So the light hole electron states may be ignored to a reasonable approximation. Under this circumstances, the Eq. 3 will become the following form:


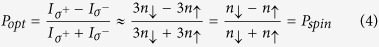


From Eq. 4 it can be seen that the Popt is likely to exceed 50% in quantum confined structure compared to the bulk material.

### Growth details

All of the samples were grown on c-plane patterned sapphire substrate (0001) using an AIXTRON 2000HT metal organic chemical vapor deposition (MOCVD) system. A 30-nm-thick GaN buffer layer was firstly grown on substrate at 540 °C. Then a 2-μm-thick undoped GaN bulk layer was grown at 1030 °C. The active layer consists of an InGaN QW layer and/or an InGaN QDs layer as well as their/its adjacent GaN barrier. The InGaN QW and QDs were grown at 740 °C. The InGaN QDs were grown by a growth interruption method^37^,^38^,^39^, which includes an initial 1.5-nm InGaN thin film growth and a subsequent 20-s growth interruption at 650 °C. The GaN barriers adjacent to QW and QDs were grown at 740 °C.

### PL and TRPL test

For the PL measurements, the 405 nm InGaN laser diode was employed as the optical excitation source. And a TRIAX 550 monochromator followed by a photomultiplier tube (PMT) were used to collect luminescence signal and detect it. The analyzer behind the laser was used to produce a linearly polarized light. The 405-/473-nm QWP was used to produce a circularly polarized light. The angle between the transmission axis of the analyzer and the fast or slow axis of the QWP is 45° degrees. And the first 532-nm QWP was used to change the circularly polarized light into the linearly polarized light, then an analyzer could detect the two linearly polarized light. The second 532-nm QWP is used in front of the monochromator to convert the linearly polarized lights into a circularly polarized light, whose fast or slow axis keeps 45^o^ degrees angle with the transmission axis of the analyzer.

TRPL spectra were measured using a 399-nm picosecond pulsed laser diode as the optical excitation source. An iHR-320 monochromator followed by a R3809U-50 microchannel plate photomultiplier tube (MCP-PMT) were used to collect luminescence signal and detect it. The testing range is 30 ns with a step size of 4 ps. The repetition rate of the laser was reduced to 2.5 MHz. Instrument response function has a half-width about 80 ps recorded at scattering light wavelength 399 nm.

## Additional Information

**How to cite this article**: Yu, J. *et al.* Study on spin and optical polarization in a coupled InGaN/GaN quantum well and quantum dots structure. *Sci. Rep.* **6**, 35597; doi: 10.1038/srep35597 (2016).

## Supplementary Material

Supplementary Information

## Figures and Tables

**Figure 1:**
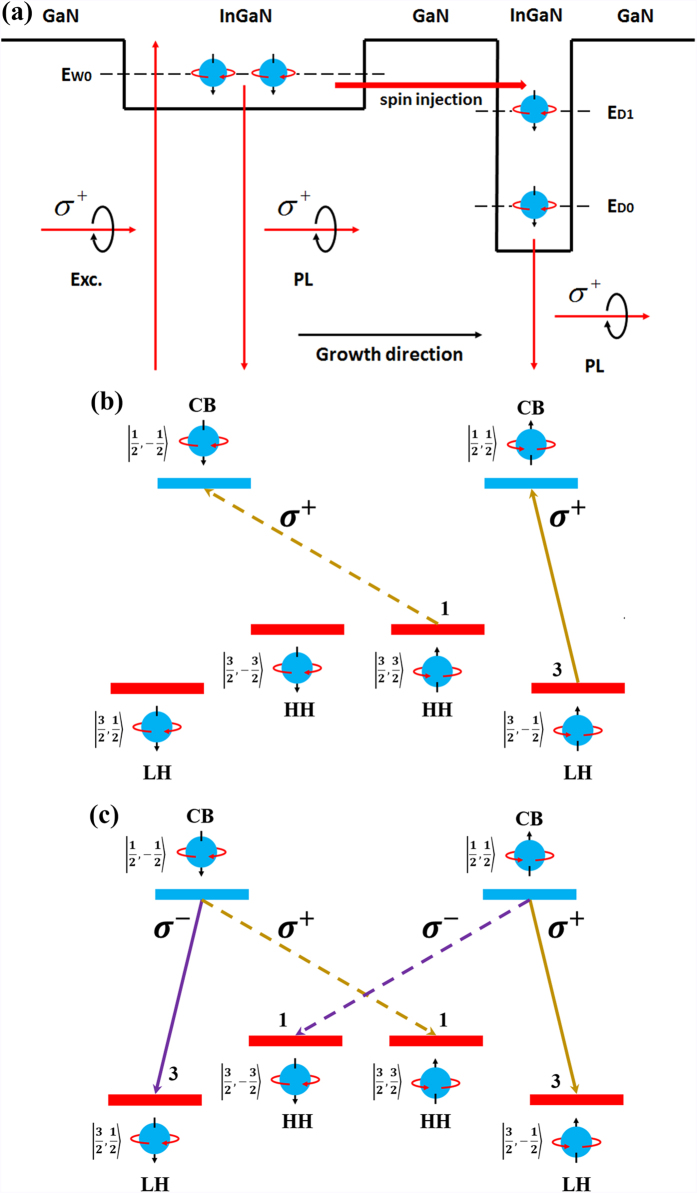
(**a**) Schematic drawing of electron-spin injection via phonon assisted tunneling in coupled InGaN QW-QDs structure with thin GaN barrier. Selection rules allowed radiative interband transitions of (**b**) InGaN QW and (**c**) InGaN QDs in which epitaxial strain and quantum confinement have lifted the heavy- and light-hole band degeneracy.

**Figure 2:**
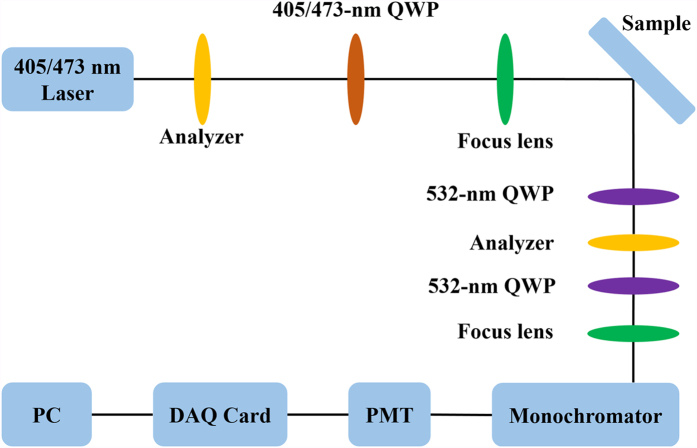
Schematic drawing of the PL test system. The details can be found in **Methods**.

**Table 1:** Specification of the conduction and valence band states near the Γ-point.

Band	*|J, m*_*j*_*〉*	Wavefunction
**Conduction band**	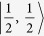 **CB**↑	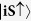
	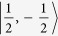 **CB**↓	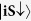
**Heavy hole band**	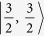 **HH**↑	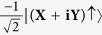
	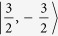 **HH**↓	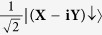
**Light hole band**	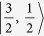 **LH**↑	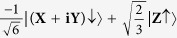
	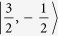 **LH**↓	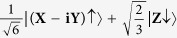

**Table 2:** The transition probabilities and the polarization state of generating/absorbing photon along the +z direction.

Transition	Δ*m*_*j*_	Emittered/Absorbed Photon	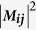
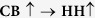	**+1**	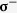	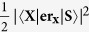
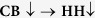	**−1**	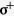	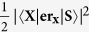
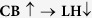	**−1**	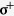	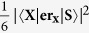
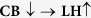	**+1**	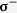	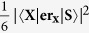
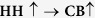	**−1**	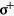	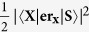
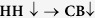	**+1**	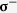	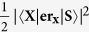
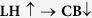	**+1**	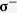	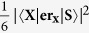
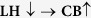	**−1**	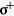	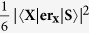
