# Supplementary material for: Study on spin and optical polarization in a coupled InGaN/GaN quantum well and quantum dots structure
Source: Sci Rep. 2016 Oct 19;6:35597. doi: 10.1038/srep35597 (PMC5069675; doi:10.1038/srep35597)
Supplement: Supplementary Information [file srep35597-s1.pdf]

Supplementary Information

Study on spin and optical polarization in a coupled InGaN/GaN quantum well and quantum dots structure

Jiadong Yu[1], Lai Wang[1]*, Di Yang[1], Jiyuan Zheng[1], Yuchen Xing[1], Zhibiao Hao[1], Yi Luo[1]*, Changzheng Sun[1], Yanjun Han[1], Bing Xiong[1], Jian Wang[1] and Hongtao Li[1]

[1]Tsinghua National Laboratory for Information Science and Technology, Department of Electronic Engineering, Tsinghua University, Beijing 100084, China.

Correspondence and requests for materials should be addressed to L.W. (email: wanglai@mail.tsinghua.edu.cn) or Y.L. (email: luoy@mail.tsinghua.edu.cn)

In order to demonstrate the polarization field in sample A is weaker, PL test with a variety of excitation power values under 405 nm excitation has been carried out on samples A and B. The blue-shift of PL wavelength as the excitation power increasing is a result of carrier screening of polarization field [1-2]. As shown in Supplementary Fig. S1, the QDs' peak wavelength of sample A decreases more slowly with the excitation power increases, which illustrates the weaker polarization field in sample A.

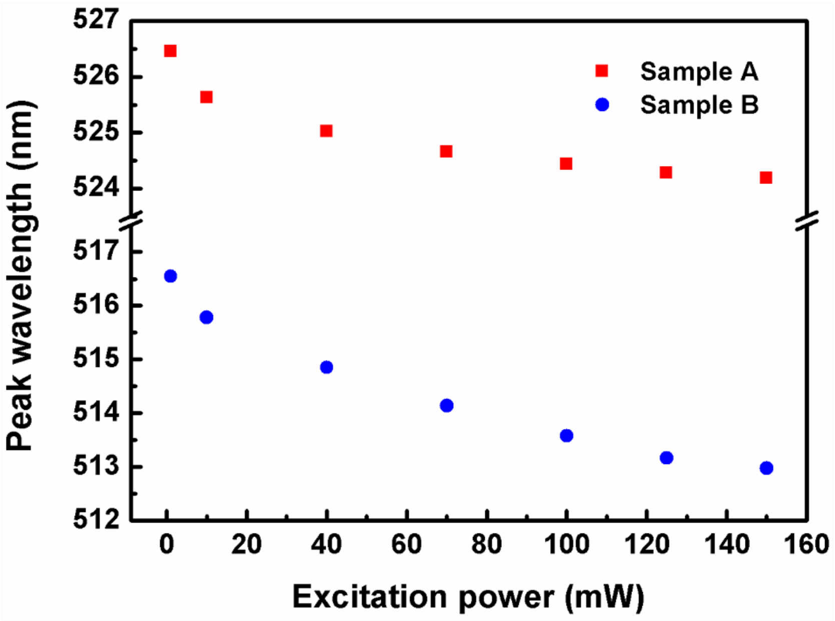

Figure S1. Dependence of QDs' peak wavelength on excitation power for samples A and B. The excitation wavelength is 405 nm.

References

[1] Kuroda, T. & Tackeuchi, A., Influence of free carrier screening on the luminescence energy shift and carrier lifetime of InGaN quantum wells. *J Appl Phys* 92 3071 (2002).

[2] Qi, Y. D. *et al.*, Comparison of blue and green InGaN/GaN multiple-quantum-well light-emitting diodes grown by metalorganic vapor phase epitaxy. *Appl Phys Lett* **86** 101903 (2005).
